# Enhancing Multi-Spectral Fingerprint Sensing for Trace Explosive Molecules with All-Silicon Metasurfaces

**DOI:** 10.3390/nano14090738

**Published:** 2024-04-23

**Authors:** Jie Lin, Ying Xue, Weijin Wang, Mingjun Sun, Shengnan Shi, Shan Zhang, Yanpeng Shi

**Affiliations:** School of Integrated Circuits, Shandong University, Jinan 250100, China

**Keywords:** fingerprint sensing, terahertz, mid-infrared, angle-scanning strategy

## Abstract

Spectroscopy is a powerful tool to identify the specific fingerprints of analytes in a label-free way. However, conventional sensing methods face unavoidable barriers in analyzing trace-amount target molecules due to the difficulties of enhancing the broadband molecular absorption. Here, we propose a sensing scheme to achieve strong fingerprint absorption based on the angular-scanning strategy on an all-silicon metasurface. By integrating the mid-infrared and terahertz sensing units into a single metasurface, the sensor can efficiently identify 2,4-DNT with high sensitivity. The results reveal that the fingerprint peak in the enhanced fingerprint spectrum is formed by the linked envelope. It exhibits a significant enhancement factor exceeding 64-fold in the terahertz region and more than 55-fold in the mid-infrared region. Particularly, the corresponding identification limit of 2,4-DNT is 1.32 µg cm^−2^, respectively. Our study will provide a novel research idea in identifying trace-amount explosives and advance practical applications of absorption spectroscopy enhancement identification in civil and military security industries.

## 1. Introduction

Terahertz (THz) and mid-infrared (mid-IR) spectroscopy are both capable of detecting and identifying many kinds of molecules with rapid and non-destructive sensing. The principle is to utilize the specific fingerprints of molecules or macromolecules ranging from mid-IR to THz bands for the quick identification of microorganisms or 2D materials [1,2]. These diverse fingerprint absorption spectra are induced by various vibrational and rotational modes of biochemical molecules [3,4]. In conventional sensing, the large mismatch between the THz or the mid-IR wavelength and the absorption cross-section of the analytes results in vibrational absorption signals that are too weak for identifying target molecules [5,6,7]. Therefore, a large quantity of samples is needed to observe significant fingerprint absorption signals [8,9,10,11]. In the past few years, to enhance the detection sensitivity and achieve the identification of trace analytes, metasurface structures have been introduced, such as metal gratings, split-ring resonators, dielectric metasurfaces, nano-antenna arrays, and waveguide structures [12,13,14,15,16,17,18,19,20,21]. These additional structures aim to amplify the electromagnetic interaction between the electromagnetic waves and analyte samples, thereby magnifying the fingerprint absorption of the analytes. Nevertheless, most of the current high-sensitivity metasurface sensors utilize a mechanism in which the sharp resonance peaks of high-quality (Q) structures change with the refractive index of the analytes [22,23,24,25]. These high-Q structures amplify electromagnetic wave–substance interaction only at isolated frequency points, which lack the capability to distinguish the broad characteristic absorption spectra of trace analytes.

Recently, a new angle-scanning strategy based on the guided-mode resonance (GMR) has been introduced in metasurface structures to achieve a broad bandwidth for sensing applications in the THz or mid-IR frequency bands [26,27,28]. Through utilizing this novel strategy, lactose and glucose can be identified successfully with the detection limits of 1.53 µg cm^−2^ and 1.54 µg cm^−2^ in the THz region, respectively [29]. Zhu et al., obtained the THz absorption spectra of lactose and 2,4-dinitrotoluene (2,4-DNT), with an enhancement about 25-fold [30]. In the mid-IR regime, the molecular fingerprints of cubic boron nitride, perfluoropolyether, and hexagonal boron nitride are amplified approximately 5.4-fold, 8.6-fold, and 34-fold, respectively [31]. Despite the fingerprints of most organics, biomolecules, and explosives being in the THz region, many proteins and antibodies have similar properties in the mid-IR range. However, the studies on this subject are limited to detecting the fingerprint of molecules within a single frequency range.

Multi-spectral metasurfaces have been widely used in other fields, such as absorbers, resonators, and filters [32]. For example, Man-junath et al., presented a single dual-region metasurface based on meander line resonators, which can interact with near-infrared and THz waves at the same time [33]. Grant et al., reported a synthetic multi-spectral metasurface achieved through hybridizing optical plasmonic filtering with mid-IR and THz absorption, thereby manipulating radiation in multiple regions simultaneously [34]. To the best of our knowledge, there has been no demonstration to date of a single metasurface with fingerprint sensing in dual spectral regions. Molecules, such as lactose, may exhibit a higher extinction coefficient in the THz region, while other analytes, such as 2,4-DNT, may display more noticeable absorption peaks in the mid-IR region [35,36,37]. Therefore, there is still a significant demand for a metasurface sensor capable of fingerprint sensing across multiple spectral ranges simultaneously.

In this paper, a multi-spectral all-silicon metasurface sensor based on an angle-scanning strategy is proposed, capable of operating in two distinct spectral regimes simultaneously, namely the THz and mid-IR ranges. The detection of trace 2,4-DNT is of great interest to the civil and military security industries because 2,4-DNT is a decomposition product of TNT with a higher vapor pressure and has been widely used for detecting explosives [38,39,40]. Through manipulation of the incident angle on the metasurface, broad envelope curves are obtained, encompassing the absorption resonances of 2,4-DNT in both regions. Compared to conventional approaches, the fingerprint peak value of the fingerprint is significantly increased, with peak enhancement reaching approximately 64-fold in the THz range and 55-fold in the mid-IR range. The designed metasurface structure exhibits great potential for enhancing the absorption spectra of trace explosive molecules in both regions, enabling the detection of trace explosive molecules. By selecting the optimal sensing bands based on the distinctive absorption characteristics of the analytes, it can detect trace samples with a highly enhanced fingerprint sensitivity. Moreover, the proposed all-silicon metasurface can be fabricated through mask photolithography and deep reactive ion etching [41].

## 2. Design and Simulation Method

**Figure 1:**
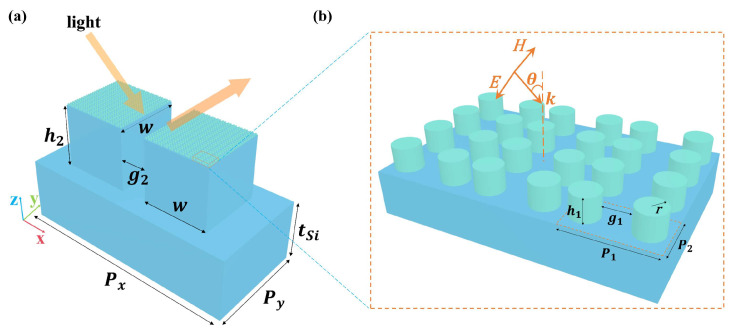
Structural diagram of the all-silicon metasurface. (**a**) The structure of the unit cell, which comprises a silicon cylinder dimer array, symmetrical silicon cubes, and a silicon substrate from top to bottom. (**b**) The inset depicts the geometrical parameters of the silicon cylinder dimer array.

Figure 1 illustrates the structural diagram of an all-silicon metasurface, comprising a silicon cylinder dimer array, symmetrical silicon cubes, and a silicon substrate from top to bottom. In both the THz and mid-IR regions, the refractive index of silicon is $n_{Si}=3.42$, and the silicon is assumed to be optically lossless and nonmagnetic [10,42]. The light (THz wave or mid-IR wave) with x-polarization is incident on the metasurface with the incident angle θ. The geometric parameters of the unit cell are demonstrated in Figure 1a, with period $P_{x}=120$ μm and $P_{y}=$56 μm, the thickness of substrate $t_{Si}=34$ μm, the height of the silicon cube $h_{2}=34$ μm, the width w=36 μm, and the gap of the two cubes $g_{2}=14$ μm. Figure 1b depicts the silicon cylinder dimer array. The structural parameters of silicon cylinder dimer array are $P_{1}=3$ μm, $P_{2}=1.5$ μm, $h_{1}=0.775$ μm, r=0.455 μm, and $g_{1}=0.87$ μm.

In order to investigate the performance of the metasensor, extensive numerical simulations were carried out using 3D simulation models with the assistance of the finite difference time domain (FDTD). Simulations on one unit cell in the THz and mid-IR ranges were performed independently by varying the incident angle. In both regions, the x and y directions were set as the periodic boundary conditions (PBC), and perfectly matched layers (PML) were imposed in the z boundaries to absorb the propagating waves. The mesh resolution was set to 4 under the auto-nonuniform type in FDTD simulation, with a minimum meshing step of 2.5 nm. The light vector k was in the x–z plane.

## 3. Results and Discussion

### 3.1. Fingerprint Detection in the THz Region

**Figure 2:**
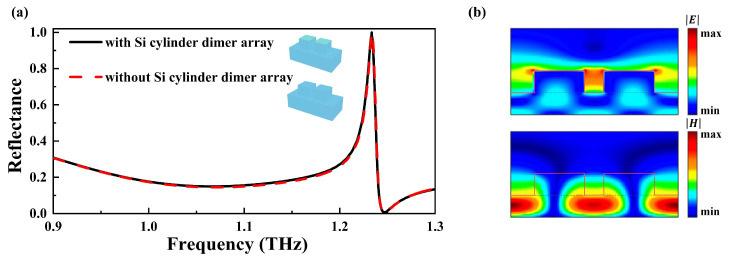
(**a**) Comparison of THz reflectance spectra of the proposed metasurface for different top layers of unit cells: the solid line is the metasurface with the silicon cylinder dimer array, and the dashed line is the metasurface without the silicon cylinder dimer array. (**b**) Cross section of the electromagnetic field profiles of the proposed metasurface at resonant frequency as observed in the x–z plane.

The numerically calculated reflectance spectra in the THz range are shown in Figure 2a, including the cases of the metasurface with and without the silicon cylinder dimer array. It can be observed that the resonant frequency response for the two samples is nearly the same, indicating that the reflectance properties in the THz region are not dependent on the geometric parameters of the silicon cylinder dimer array. A high Q resonant peak can be seen located at 1.23 THz (the solid line) in the reflectance spectrum. The full width at half maximum (FWHM) of the reflectance resonance is about 14 GHz, and the Q factor of this resonance is about 88. To gain a better understanding of the resonance mechanism, electric field distribution and magnetic field distribution at the resonance frequency of 1.23 THz were analyzed in the x–z cross section. As demonstrated in Figure 2b, an enhanced electric field is concentrated within the two silicon cubes, while the magnetic field exhibits pronounced localization and confinement in the silicon substrate layer. It is evident that the metasurface enhances the local electric and magnetic fields significantly, which can strengthen the interaction between the incident THz wave and analytes.

**Figure 3:**
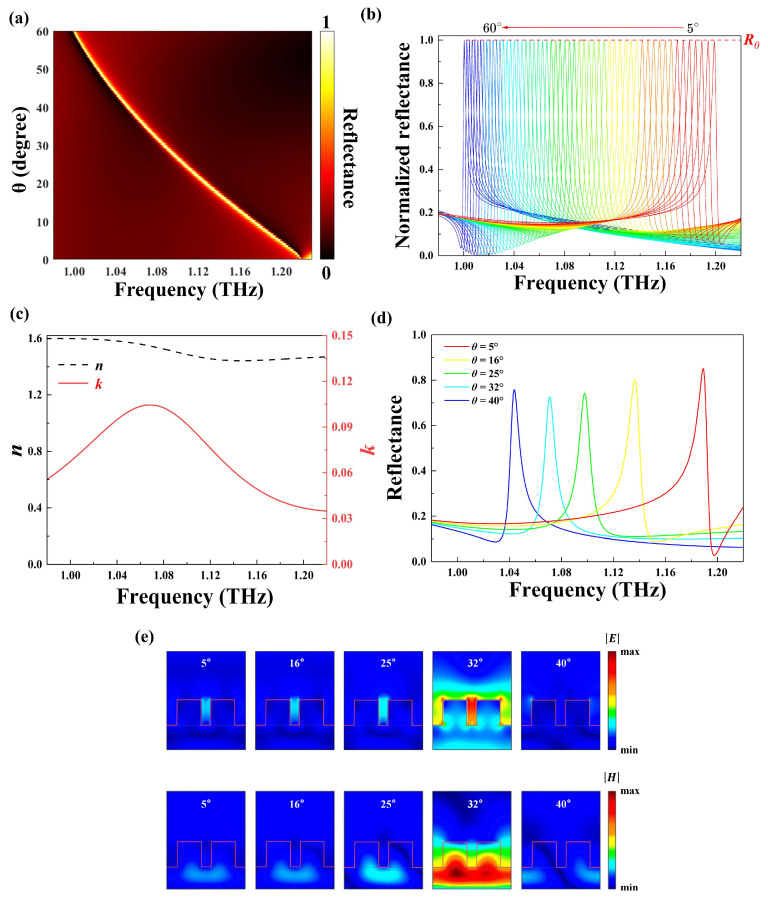
(**a**) A two-dimensional contour map of reflectance as a function of the incident angle and frequency. (**b**) Normalized reflectance spectra (incident angle ranging from 5° to 60°) without the analyte. The red dashed line is the envelope curve of the spectra. (**c**) The complex refractive index of 2,4-DNT in the THz region. (**d**) The angle-dependent reflectance spectra of 0.5 μm thick 2,4-DNT on the metasurface. (**e**) Field distributions of the 2,4-DNT film-coated dielectric metasurface at 1.07 THz with different incident angles.

In order to gain insight into the physical nature, the angle-dependent THz response of the structure was investigated. The reflectance as a function of the incident angle θ for the metasurface is plotted in Figure 3a, which indicates multiple narrow reflectance bands. It can be seen that there is a narrowband unity reflectance corresponding to each incident angle and that the frequency of maximum reflectance has a linear monotone decrease as the incident angle enlarges from 5° to 60°. Such a THz response can be interpreted by the theory of GMR, in which an incident light enters the grating waveguide at a specific angle and forms a resonance mode at the corresponding frequency [43,44,45]. In this mode, the light can be trapped and guided within the grating waveguide. Within our structure, the silicon cubes correspond to the gratings, with the silicon substrate serving as the waveguide layer. In this case, the incident THz wave was coupled to the waveguide mode through diffraction and thus excited the GMR, indicating that the high Q resonant peak located at 1.23 THz shown in Figure 2a is the GMR. The central frequency and bandwidth of the GMR depend on geometric parameters, such as the period, the thickness of the unit cell, and the gap between the two silicon cubes. This means that the structure has a high tolerance for manufacturing processes. As presented in Figure 3b, the incident THz waves (θ from 5° to 60° with a step of 1°) are regulated to form a series of sharp resonances. The resonance frequency shifts from 1 to 1.2 THz, which covers the absorption band of 2,4-DNT in the THz regime. To obtain the absorbance spectra of the analyte, the resonance peaks are first calculated and then fitted into an envelope curve (illustrated by the red dashed line) using interpolation. After the metasurface structure is covered with the analyte, an absorption peak corresponding to the unique fingerprint of the analyte emerges within the envelope curve.

The sensing performance was evaluated comprehensively with a 2,4-DNT film coated on the metasurface. Figure 3c shows the 2,4-DNT’s complex refractive index [46,47,48]. It was found that the refractive index of 2,4-DNT undergoes a significant decrease around 1.07 THz. Simultaneously, its extinction coefficient reaches its peak values at 1.07 THz, corresponding to the central frequency of the THz absorption bands. To illustrate molecular fingerprint detection, a 0.5 μm thick layer of 2,4-DNT was coated on the metasurface, as shown in Figure 3d. With the increasing of incident angles (5°, 16°, 25°, 32°, and 40°), the reflectance peak undergoes a remarkable red shift while the peak amplitude declines from 85.08% to 72.58% first and then increases to 75.74%. The resonance peak reaches a minimum value at 1.07 THz for an incident angle of 32°, which corresponds to the maximum of the extinction coefficient curve for 2,4-DNT. The electric and magnetic field distributions for different incident angles at the fixed frequency of 1.07 THz is depicted in Figure 3e. As the incident angle gradually changes from 5° to 40°, a significant electric and magnetic field enhancement appears at 1.07 THz for θ = 32°. The magnetic field is mainly confined in the wave-guided layer, and a strong electric field is induced between the two silicon cubes. However, the electric and magnetic fields are greatly reduced at other incident angles. This is because the resonance frequency of these incident angles is far from the peak value frequency of the extinction coefficient. Such electric field distribution could enhance wave–matter interaction around the resonant frequency, enhancing the sensing capability of the metasurface.

**Figure 4:**
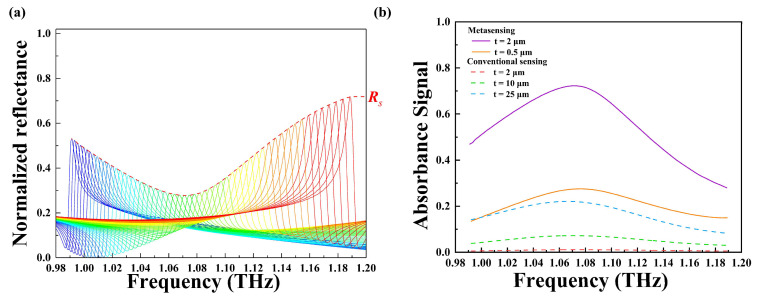
(**a**) Normalized reflectance spectra (the angle of the incident source ranging from 5° to 60°) and their corresponding envelope curve (the dashed red line) for the metasurface with a 2 μm thick 2,4-DNT layer coating. (**b**) Absorbance signals of different thickness of 2,4-DNT with (the solid line) and without (the dashed line) the metasurface.

Figure 4a shows a series of normalized reflectance spectra acquired with the incident angle scanning from 5° to 60° (one reflectance spectrum for each angle). An envelope curve (the dashed line in red) is obtained by linking all the peak values of the reflectance spectra, with a dip around 1.07 THz. In order to quantify the retrieved absorbance features of trace analytes, the absorbance signal ($A_{s}$) can be calculated from the following equation:

(1) $$A_{s}=1-R_{s}/R_{0}$$

where $R_{0}$ and $R_{s}$ are the envelope of peak reflectance without and with 2,4-DNT, respectively.

The absorbance signal (depicted in purple) plotted in Figure 4b closely resembles the extinction coefficient curve of 2,4-DNT depicted in Figure 3c, indicating that the fingerprint sensor based on an all-silicon metasurface can accurately identify 2,4-DNT. The absorbance signals of the metasensing are presented in Figure 4b for different thicknesses (0.5, 2 μm) of 2,4-DNT. Additionally, the absorbance spectra of 2,4-DNT for conventional sensing are plotted for comparison. In the conventional sensing method, the analyte is placed on an unpatterned silicon substrate with a thickness of 34 μm, and the absorbance signal is obtained under normal incidence. The absorbance spectrum for the 2 μm thick 2,4-DNT obtained by conventional sensing is less than 1.19%, and its molecular fingerprint cannot be identified. Although the thickness increases to 10 μm, the conventional sensing signal intensity is extremely low, with an absorbance peak value of about 7%. In contrast, it can be observed that the detection signal intensity of the 2 μm thick 2,4-DNT obtained by metasensing is enhanced from 1.12% to 72.22% at 1.07 THz, which demonstrates about a 64-fold enhancement. It is worth noting that even with a minimal thickness of 0.05 μm, 2,4-DNT can be detected successfully with an absorbance peak value of about 27.52%, which is even larger than that of a 50 μm thick sample obtained by conventional detection.

### 3.2. Fingerprint Identification in the Mid-IR Region

**Figure 5:**
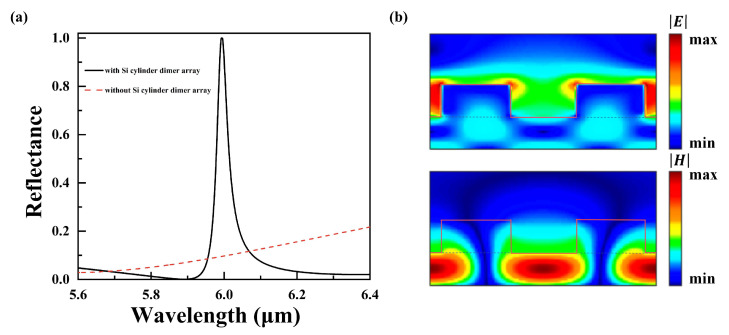
(**a**) Reflectance spectra of the proposed metasurface in the mid-IR region: the solid line is the metasurface with the silicon cylinder dimer array and the dashed line is the metasurface without the silicon cylinder dimer array. (**b**) Cross section of the electromagnetic field profiles of the proposed metasurface at the resonant wavelength as observed in the x–z plane.

After the fingerprint sensing ability of the proposed metasurface in the THz range was demonstrated, its mid-IR resonance properties were investigated in detail. The mid-IR reflectance spectra in the wavelength ranging from 5.6 to 6.4 μm are depicted in Figure 5a. This shows that the resonant wavelength is 5.99 μm when mid-IR waves irradiate the metasurface vertically. The resonance peak vanishes for the metasurface without the silicon cylinder dimer array. The FWHM of the resonance is about 0.0368 μm, and the Q factor can reach 163, indicating that the metasurface has an extremely high Q value in both bands. Figure 5b shows the electric and magnetic field profiles at the fixed wavelength of 5.99 μm. At the resonance, the electric field surrounding the silicon cylinder dimer array shows a significant enhancement. Silicon cubes (regarded as substrates) under the silicon cylinder dimer array are considered as the waveguide layer where the magnetic field is strongly localized and confined. It can be observed that the enhancement of the electromagnetic field in the mid-IR band is similar to that in the THz region. Hence, the fingerprint detection capability of trace amounts of analytes has been significantly enhanced.

**Figure 6:**
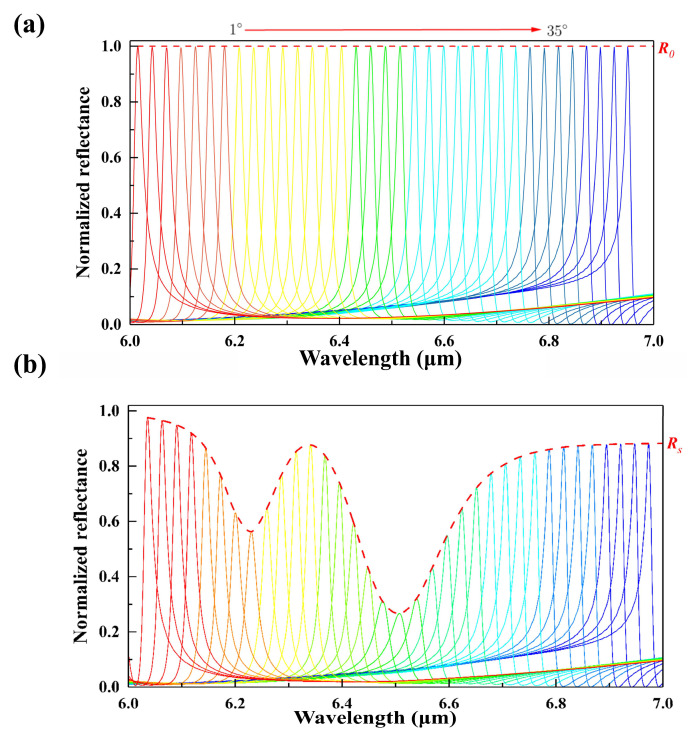
(**a**) Normalized reflectance spectra without 2,4-DNT coatings and the corresponding envelope curve (the dashed red line). (**b**) Normalized reflectance spectra coated with 20 nm 2,4-DNT and their corresponding envelope curve (the dashed red line).

As shown in Figure 6a, the incident angle of mid-IR waves (from 1° to 35°) operates to form a series of sharp resonance peaks so that the characteristic absorption bands of 2,4-DNT in mid-IR range are well covered by the normalized reflectance spectra. The complex dielectric permittivity of 2,4-DNT in the mid-IR regime is based on a Drude–Lorentz oscillator model [39,49]. The absorption resonance of 2,4-DNT from 6.0 to 7.0 μm is considered in the present study. Therefore, the complex dielectric permittivity of 2,4-DNT can be calculated from the following equation:

(2) $$\varepsilon_{r}\left(\omega \right)=\varepsilon_{\infty }+\sum_{n=1}^{4}A_{n}\frac{\omega_{n}^{2}}{\omega_{n}^{2}-\omega^{2}-i\gamma_{n}\omega }$$

where $\varepsilon_{r}\left(\omega \right)$ is the permittivity, $\omega_{n}$ is the resonant frequency, the background of 2,4-DNT is $\varepsilon_{\infty }$= 2.18, $\lambda_{n}$ is the wavelength, $\gamma_{n}$ is the damping factor, and $A_{n}$ is the absorbed amplitude of each oscillator. The parameters chosen are shown in Table 1.

The complex refractive index of 2,4-DNT can be calculated from the following equation:

(3) $$N\left(\omega \right)=\sqrt{\varepsilon_{r}\left(\omega \right)}=n\left(\omega \right)+ik\left(\omega \right)$$

where $n\left(\omega \right)$ represents the refractive index, and $k\left(\omega \right)$ is the extinction coefficient. When a 20 nm thick layer of 2,4-DNT is physically coated on the metasurface, the fingerprint absorption signals are revealed by the envelope of normalized reflectance amplitude show in Figure 6b. It is clear that the normalized spectra with 2,4-DNT coating have a remarkable attenuation, which is caused by the absorption characteristics of the 2,4-DNT.

**Figure 7:**
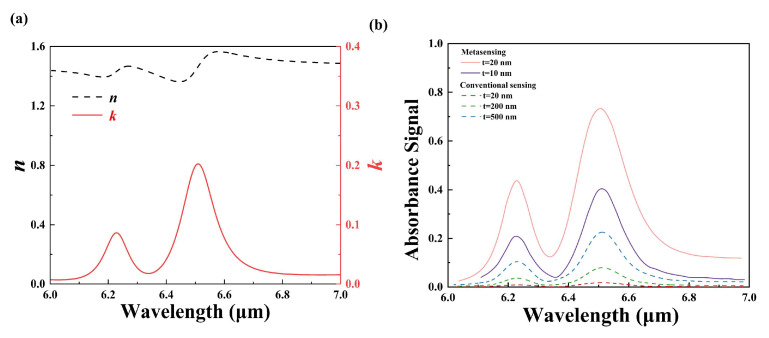
(**a**) The calculated complex refractive index of 2,4-DNT in the mid-IR region. (**b**) Absorbance signals of different thicknesses of 2,4-DNT with (the solid line) and without (the dashed line) the metasurface.

Figure 7a displays the complex refractive index of 2,4-DNT from 6.0 to 7.0 μm, which is calculated by Equation (3). The extinction coefficient of 2,4-DNT in mid-IR ranges exhibits two obvious peak features at the wavelengths of about 6.23 and 6.51 μm. The absorption fingerprints of 2,4-DNT molecules in mid-IR region are revealed by calculating the absorbance signal as shown in Figure 7b. The amplitude of the reference absorbance by conventional sensing increases with the 2,4-DNT thickness. As the thickness increases from 20 nm to 200 and to 500 nm, the corresponding absorbance peak values at the resonant wavelength of 6.51 μm are 1.72%, 7.93%, and 22.5%. In contrast, the envelope signal intensities obtained by metasensing for 20 nm thick 2, 4-DNT greatly enhances the two fingerprint points, increasing extraordinarily the peak values from 0.793% and 1.72% to 43.72% and 73.36%, respectively. The absorbance signal is enhanced by nearly 55-fold and 42.7-fold. The potential of the platform in explosive analysis and fingerprint identification was demonstrated by comparing its sensing ability with and without 2,4-DNT molecules. The detection limit (σ) can be calculated using the following equation:

(4) σ=ρ×h

where h is the thickness, and ρ is the crystal density of 2,4-DNT. In our study, the crystal density of 2,4-DNT is ρ=1.32 µg cm^−3^, and the minimum thickness of 2,4-DNT that can be detected is 10 nm; hence, the detection limit of the proposed metasurface for 2,4-DNT detection is calculated to be 1.32 µg cm^−2^.

Finally, to illustrate the novelty and significance of the proposed metasurfaces, comparisons between the previous fingerprint metasensors and the designed structure in this paper are listed in Table 2. It is obvious that our dual-region metasurface device demonstrates the capability to achieve high-sensitivity fingerprint detection in the mid-IR and THz bands.

## 4. Conclusions

In summary, a multi-spectral fingerprint detection scheme based on angle-scanning sensing is proposed, which can operate simultaneously in the mid-IR and THz regions. The metasurface incorporates silicon cylinder dimer arrays into THz unit cells on the microscale. The presented electromagnetic field distributions at reflectance peaks reveal a similar GMR in both regions with a high Q factor, which can be induced when the lights are vertically incident on this metasurface. By employing the angle-scanning strategy, the metasurface can identify 2,4-DNT through absorbance spectra, and a wide absorbance signal envelope curve that covers a wide range of frequencies in two different regime is obtained. Our design has significant potential for the fingerprint detection of trace amounts of analytes in both regions, which will facilitate many new applications on non-destructive analysis. Additionally, the successful fingerprint detection of explosive molecules will meet the requirements of both civil and military security industries.

## Figures and Tables

**Table 1:** The parameters for the Lorentz model of permittivity of 2,4-DNT.

n	$\omega_{n}\left(cm^{-1}\right)$	$\lambda_{n}\left(\mu m\right)$	$\gamma_{n}\left(cm^{-1}\right)$	$A_{n}$
1	1610	6.21	15	1.68 × 10^−3^
2	1603	6.24	15	7.8 × 10^−4^
3	1538	6.50	22	7.4 × 10^−3^
4	1522	6.57	23	3.1 × 10^−3^

**Table 2:** The list for the fingerprint detection performances of the metasensors.

Ref.	Material of Structured Layer	Analyte	Multiplexed Scheme	Working Range	Peak Enhancement Time
[26]	Silicon	Protein A/G	Geometry	Mid-IR	~60
[27]	Germanium	PMMA	Angle	Mid-IR	~50
[30]	PE	α-lactose	Angle	THz	~25
[5]	ABS	α-lactose	Angle	THz	~45
This work	Silicon	2,4-DNT	Angle	Mid-IR/THz	~55/~64

## Data Availability

Data underlying the results presented in this paper are not publicly available at this time but may be obtained from the authors upon reasonable request.
